# Impact of intravenous push antibiotics on sepsis management at a Veterans Affairs medical center emergency department

**DOI:** 10.1017/ash.2026.10761

**Published:** 2026-07-09

**Authors:** Mercy Hoang-Nguyen, Kang Lim, Catherine Vo, Luis Tulloch-Palomino

**Affiliations:** 1 Pharmacy, VA Puget Sound Health Care System Seattle Division, USA; 2 VA Puget Sound Health Care System, USA; 3 Hospital & Specialty Medicine, VA Puget Sound Health Care System, USA; 4 Division of Allergy and Infectious Disease, Department of Medicine, University of Washington School of Medicine, Seattle, WA, USA

## Abstract

**Objective:**

To detail the impact of transitioning from intravenous piggyback (IVPB) to intravenous push (IVP) antibiotics on median time from sepsis identification to antibiotic administration (minutes), proportion of septic patients who received antibiotics within 60 and 180 minutes of sepsis identification, and Emergency Department (ED) length of stay (LOS).

**Design:**

Retrospective chart review.

**Setting:**

Urban, acute-care hospital.

**Participants:**

394 patients received IVPB antibiotics (June 2022–June 2023) and 421 patients received IVP antibiotics (June 2023–June 2024), identified through sepsis-related ICD-10 codes, systemic inflammatory response syndrome (SIRS) criteria, and other clinical indicators.

**Methods:**

Chart reviews were conducted to obtain sepsis identification and antibiotic administration start times. Statistical process control was used to monitor trends and assess process consistency over time.

**Results:**

Following our intervention, median time from sepsis identification to antibiotic administration decreased from 132 minutes (IQR 70, 194) in the IVPB group to 99 minutes (IQR 56,164) in the IVP group (*P* < .001). The proportion of patients who received antibiotics within 60 minutes of sepsis identification increased from 20.3% to 28.0% (*P* = .01) and within 180 minutes increased from 70.8% to 79.8% (*P* = .003). ED LOS decreased from 397 minutes with IVPB to 369 minutes with IVP (*P* = .004). A sustained downward shift of twelve consecutive months of mean sepsis identification to antibiotic administration start time below the pre-intervention centerline was observed.

**Conclusions:**

IVP antibiotics were associated with decreased median time from sepsis identification to antibiotic administration for septic patients, increased adherence to practice guidelines, and reduced ED LOS.

## Introduction

Prompt treatment of sepsis is crucial for improving patient outcomes. The 2021 and 2026 Surviving Sepsis Campaign (SSC) Guidelines recommend antibiotic administration within one hour for patients with possible septic shock or a high suspicion of sepsis, and within three hours for patients with possible sepsis without shock.^1,2^ Delays in antibiotic administration have been associated with increased morbidity and mortality in sepsis patients.^3^

Intravenous piggyback (IVPB) antibiotics require on-demand compounding or thawing from premixed frozen solution and are administered over 30–60 minutes. Admixture devices (eg, vial-to-bag adapters) increase cost, training burden, and risk of incomplete or incorrect activation leading to no medication delivery. In contrast, intravenous push (IVP) antibiotics are low cost, ready-to-use, without risk of drug omission, and can be administered over 5 minutes without affecting their pharmacodynamic profile compared to 30-minute infusion.^4,6^ Additionally, IVP antibiotics have been shown to decrease delays in antibiotic administration, labor demands, and cost without any increase in adverse events compared to IVPB antibiotics.^5–13^

Given the growing evidence supporting IVP antibiotics, our Emergency Department (ED) transitioned from IVPB to IVP administration for cefazolin, cefepime, ceftriaxone, and piperacillin-tazobactam to improve time to antibiotic administration and compliance with SSC guidelines. This retrospective analysis evaluates outcomes before and after the transition.

## Methods

### Population and setting

We conducted a retrospective analysis of adult patients with sepsis treated in the Veterans Affairs Puget Sound Healthcare System (VAPSHCS) ED between June 2022 and July 2024. The VAPSHCS is a tertiary academic medical center with approximately 22,000 annual ED visits. Patients were included if they had a sepsis-related International Classification of Diseases (ICD)-10 code or met ≥2 systemic inflammatory response syndrome (SIRS) criteria. Exclusion criteria included receipt of antibiotics before documented sepsis identification, unclear antibiotic start times, duplicate records, or failure to meet SIRS criteria on chart review. Additional chart review captured key variables, including sepsis identification time (defined as meeting ≥2 SIRS criteria), antibiotic administration start time, ED length of stay (LOS), patient demographics, antibiotic selection, and suspected infection source.

### Intervention

Before the intervention, cefazolin, cefepime, ceftriaxone, and piperacillin-tazobactam were dispensed to the ED as IVPB, requiring on-demand compounding or thawing from pre-mixed frozen solution, and infusion over 30 minutes. In June 2023, our ED transitioned the first dose of these antibiotics to IVP administration. To support this change, antibiotic vials and diluents were stocked in the ED automated dispensing cabinet for rapid access. Education regarding the change was provided to ED physicians, nurses, and pharmacy staff. Training on immediate-use compounding was delivered to ED nurses prior to rollout and subsequently incorporated into annual nursing competency requirements. This intervention supported ongoing efforts to improve compliance with the SSC guidelines. Following the initial IVP dose in the ED, subsequent antibiotic doses continued as IVPB (30-minute infusion for cefazolin, cefepime, and ceftriaxone; extended infusion for piperacillin-tazobactam).

### Primary and secondary outcomes

The primary measure was the median time from sepsis identification to antibiotic administration start (minutes) per month. Secondary measures included the proportion of septic patients who received antibiotics within 60 and 180 minutes of sepsis identification and ED LOS.

### Statistical analysis

Descriptive and inferential statistics were used to summarize variables before and after the intervention. X-bar and S charts assessed process consistency over time and temporal relationships between IVP antibiotic implementation and mean time from sepsis identification to antibiotic administration using accepted rules for special cause variation.^14^ Comparisons between groups were performed using Wilcoxon rank sum and chi-squared analysis, as appropriate. Analyses were conducted in Stata version 18.1 (College Station, TX) and Process Improvement Products QI Charts for Microsoft Excel version 2.0 (San Antonio, TX). All tests were two-sided, and *P* < .05 was considered statistically significant.

## Results

**Figure 1.**
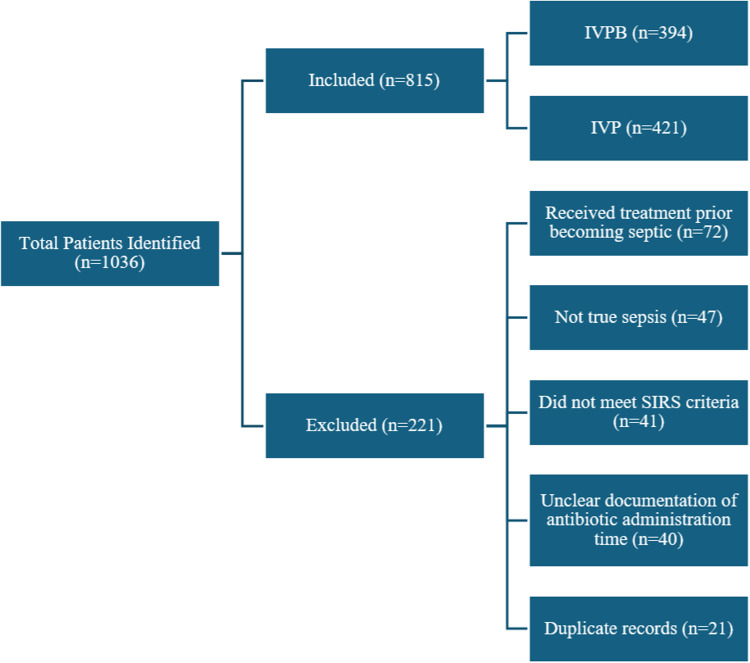
Patient selection. The Figure illustrates the patient selection process, showing the number of patients included in the IVPB and IVP groups, as well as the number of patients excluded with corresponding reasons for exclusion.

Of the 1,036 encounters identified, 221 (21%) were excluded due to duplicate records (n = 21), antibiotic administration before sepsis identification (n = 72), unclear antibiotic administration start time (n = 40), or not meeting sepsis criteria after adjudication (n = 88). We included 815 in the final analysis, with 394 (38%) receiving IVPB antibiotics and 421 (40%) receiving IVP antibiotics (Figure 1).

Baseline characteristics between the two groups were similar, with no significant difference in age between the IVPB (71 yr; IQR 62, 76) and IVP groups (71 yr; IQR 61, 77). Ceftriaxone was the most frequently used antibiotic (IVPB 59% vs IVP 63%), and pneumonia was the most frequent indication for antibiotics (IVPB 33% vs IVP 33%) in both groups (Table 1). The time from sepsis identification to antibiotic administration start was 132 (IQR 70, 194) minutes with IVPB antibiotics and 99 (IQR 56, 164) minutes with IVP antibiotics. Patients in the IVPB group were less likely to receive antibiotics within 60 (IVPB 20% vs IVP 28%) and 180 (IVPB 71% vs IVP 80%) minutes of sepsis identification. Regarding ED LOS, patients in the IVPB group stayed for a median of 397 (IQR 284, 576) minutes and patients in the IVP group stayed for a median of 369 (IQR 262, 482) minutes (Table 2).

**Table 1.** Baseline characteristics, antibiotic use, and infection indications in IVPB and IVP groups

	IVPB N = 394	IVP N = 421	*P*-value
Age (IQR)	71 (62, 76)	71 (61, 77)	.9812
Antibiotics, n (%)			
– Cefazolin	11 (2.8)	7 (1.7)	.273
– Cefepime	71 (18)	88 (21)	.299
– Ceftriaxone	232 (58.9)	266 (63.1)	.208
– Piperacillin-tazobactam	80 (20.3)	60 (14.2)	.022
Antibiotic indications, n (%)			
– Pneumonia	131 (33.2)	138 (32.8)	.887
– Urinary tract infection	95 (24.1)	88 (20.9)	.273
– Cellulitis	57 (14.5)	59 (14)	.853
– Unknown etiology/other infections	44 (11.2)	57 (13.5)	.304
– Intra-abdominal infection	38 (9.6)	47 (11.1)	.478
– Bone/joint infection	25 (6.3)	28 (6.6)	.86
– Bacteremia	4 (1)	4 (0.9)	.925

**Table 2.** Process and clinical outcomes of IVPB vs IVP antibiotic

Outcomes	IVPB N = 394	IVP N = 421	*P*-value
Median time from sepsis identification to antibiotic administration, minutes (IQR)	132 (70, 194)	99 (56, 164)	<.001
Proportion of sepsis patients received antibiotics <60 minutes, n (%)	80 (20.3%)	118 (28%)	.01
Proportion of sepsis patients received antibiotics <180 minutes, n (%)	279 (70.8%)	336 (79.8%)	.003
ED LOS, minutes (IQR)	397 (284, 576)	369 (26, 482)	.004

**Figure 2.**
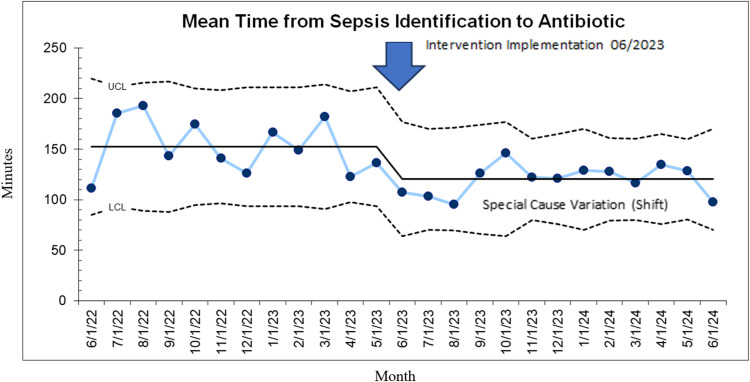
Statistical process control chart for mean time from sepsis identification to antibiotic administration. This figure displays the fluctuation in mean time from sepsis identification to antibiotic administration prior to the intervention, with some data points approaching the upper control limit (UCL). Following the implementation of IVP antibiotic administration in June 2023, the data show a sustained downward shift, with twelve consecutive months below the pre-intervention centerline and reduced month-to-month fluctuation.

**Figure 3.**
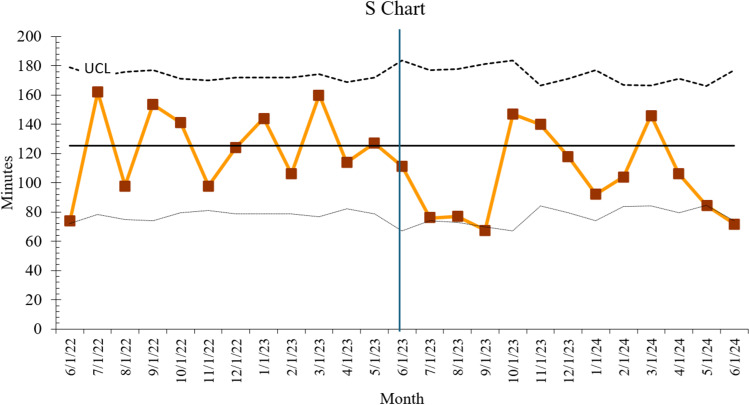
Statistical process control S chart for variability in time from sepsis identification to antibiotic administration. Prior to IVP implementation, standard deviations were higher and inconsistent, with several months showing large spikes in administration times. Post-intervention, the standard deviation trended downward, with less extreme monthly variation and values remaining more stable within control limits.

Prior to the intervention, the mean time from sepsis identification to antibiotic administration fluctuated widely between 111 and 192 minutes, with some data points approaching the upper control limit (UCL). Following implementation of IVP antibiotic administration, there was a sustained downward shift of twelve consecutive months of mean sepsis identification to antibiotic administration start time below the pre-intervention centerline and less variations across data points (Figure 2). In terms of variability, the monthly standard deviation was less consistent before the intervention, with three points beyond the control limits. After the intervention, the monthly standard deviation remained entirely within the control limits (Figure 3).

## Discussion

IVP antibiotic administration is supported by the American Society of Health-System Pharmacists (ASHP) to conserve IV fluids during shortages and has gained adoption for its practical benefits.^5^ The shortened infusion time of IVP regimens was found to have no significant impact on the pharmacodynamic profile of the antibiotics, having little to no effect on plasma concentration throughout the dosing interval or the time interval that free drug concentration remained above MIC.^6^ Additionally, IVP antibiotics can reduce labor demands, improve time to antibiotics, lower costs, and does not increase adverse events.^7–13^

The findings from this QI initiative demonstrate that the transition from IVPB to IVP antibiotics was associated with significant improvements in the timeliness and consistency of antibiotic administration for sepsis patients at the VAPSHCS ED. The median time from sepsis identification to antibiotic administration was significantly reduced from 132 minutes (IQR 70–194) with IVPB to 99 minutes (IQR 56–164) with IVP (*P* < .001). Additionally, adherence to SSC guidelines improved, as reflected by an increase in the proportion of patients receiving antibiotics within 60 minutes (20.3% to 28%, *P* = .01) and within 180 minutes (70.8% to 79.8%, *P* = .003). These findings are supported by the trend of the X-bar chart, which showed a consistent downward shift in the mean time from sepsis identification to antibiotic administration post intervention. Lastly, the ED LOS decreased by 28 minutes (397 to 369 mins, *P* = .004), which along with the improved process consistency showed by the S chart, suggests that the intervention had a modest effect on overall ED efficiency and patient flow.

These findings align with previous studies demonstrating time savings with IVP antibiotics in sepsis management. In prior retrospective analysis, IVP was associated with approximately 32-minute time savings to *β*-lactam (*β* = –0.60; 95% CI, –0.91 to –0.29) and to broad-spectrum (*β* = –0.32; 95% CI, –0.62 to –0.02) antibiotic administrations. The IVP antibiotic group was less likely to fail the goal of *β*-lactam antibiotics within 1 hour (44.6% vs 57.3%; odds ratio, 2.27; 95% CI, 1.34–3.86) and 3 hours (7.6% vs 24.5%; odds ratio, 4.31; 95% CI, 2.01–10.28) of sepsis diagnosis compared with IVPB.^11^ However, with a small sample size and short timeframes, the study did not evaluate trends in antibiotic administration times or assess process consistency over time, which is critical for QI initiatives.

Our analysis is the first to demonstrate a statistically significant association between IVP antibiotics and improvements in sepsis-related process measures and patient outcomes, using a large sample size with statistical process control evaluation over time. Despite these improvements, some challenges remain. Only 28% of patients received IVP antibiotics within 60 minutes, and although the proportion of patients receiving antibiotics within 180 minutes increased to 79.8%, approximately 20% still did not receive antibiotics within the recommended time frame, indicating a need for further optimization.

Several institution-specific factors likely contributed to lower adherence. Our analysis measured time from sepsis identification, which depends on timely clinical recognition and documentation of SIRS criteria. Variability in these processes may have attenuated measured adherence despite timely clinical intent. Limited space in ED automated dispensing cabinets also restricted the quantity of antibiotic and diluent vials that could be stocked, necessitating frequent restocking and potentially delaying antibiotic administration. Furthermore, the reduction in ED LOS, while statistically significant, may not be clinically meaningful in all cases, indicating that other factors beyond antibiotic timing may influence patient flow and disposition.

Overall, this initiative demonstrates that IVP antibiotics are an effective and sustainable strategy to reduce antibiotic administration times, improve adherence to sepsis guidelines, and enhance ED efficiency. Future directions should focus on expanding IVP protocols in the ED, addressing remaining delays, and evaluating the long-term patient outcomes such as mortality and ICU admission rates.

## Conclusion

Implementation of IVP antibiotics was associated with decreased median time to antibiotic administration for septic patients as well as increased adherence to SSC guidelines and decreased ED LOS.
